# Supplementary material for: Regulation of mature mRNA levels by RNA processing efficiency
Source: NAR Genom Bioinform. 2023 Jun 9;5(2):lqad059. doi: 10.1093/nargab/lqad059 (PMC10251645; doi:10.1093/nargab/lqad059)
Supplement: lqad059_Supplemental_File [file lqad059_supplemental_file.pdf]

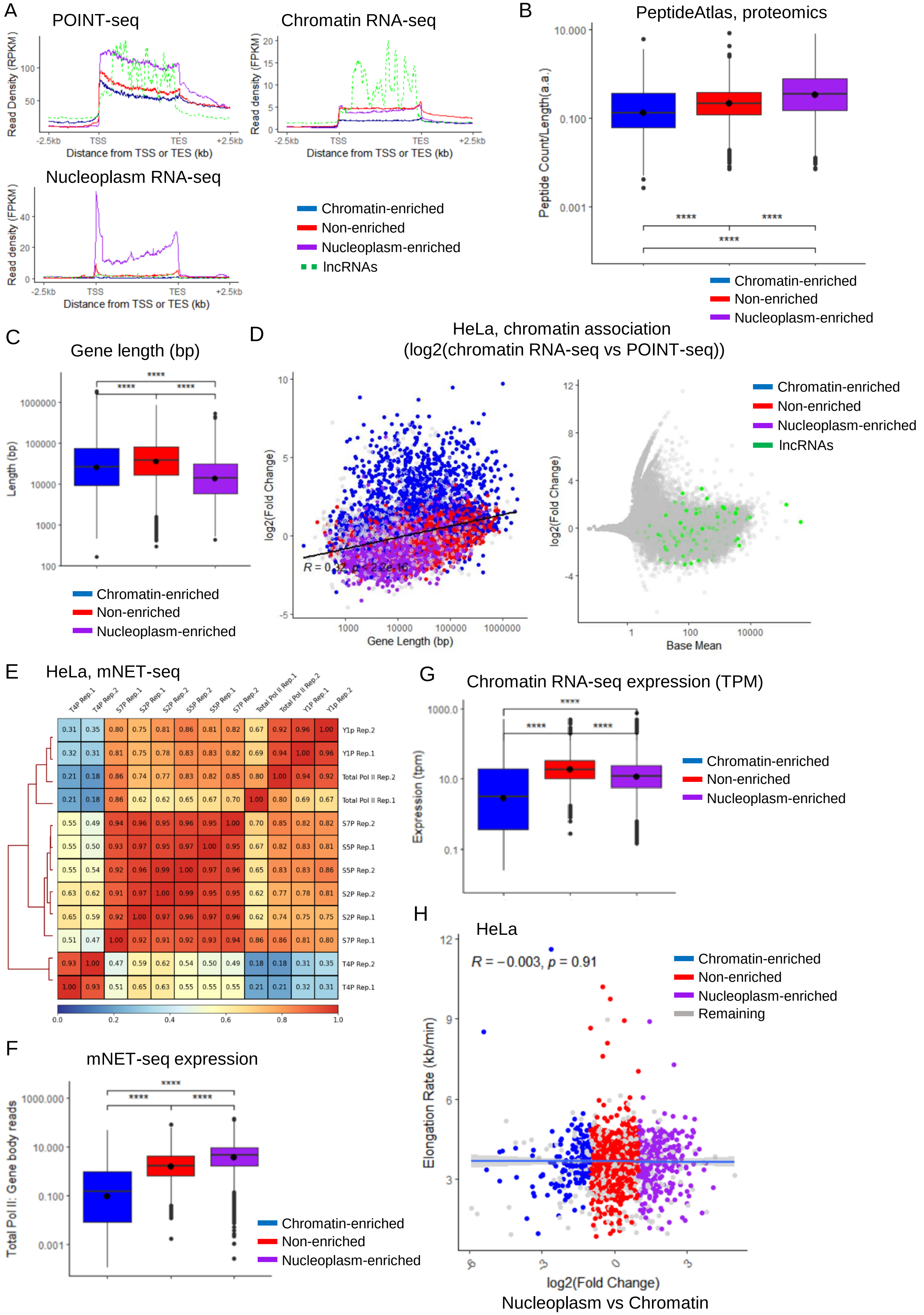

Supplementary Figure 1

Supplementary Figure 1. Higher levels of pol II Thr4 phosphorylation might be associated with poor expression and chromatin enrichment of transcripts.

(A) POINT-seq, chromatin RNA-seq, and nucleoplasm RNA-seq metagene profiles in HeLa cells of nucleoplasm-enriched (purple), chromatin-enriched (blue), non-enriched (red) genes, or lncRNAs (green). **(B)** Boxplots, shown as min to max with first quartile, median, and third quartile, of the number of peptides per protein found in the PeptideAtlas database for the nucleoplasm-enriched (purple), chromatin-enriched (blue), or non-enriched (red) genes. The number of proteins found to have at least one peptide are indicated at the top of each category. Statistical test: Wilcoxon rank sum test. P-value: **** < 0.0001. **(C)** Boxplots, shown as min to max with first quartile, median, and third quartile, of the gene length of the nucleoplasm-enriched (purple), chromatin-enriched (blue), or non-enriched (red) genes. **(D)** Left: XY correlation plot of the gene length (bp) versus the log2 fold change of “Chromatin RNA-seq versus POINT-seq”, which provides a measure of chromatin association. The Pearson correlation with p-value is indicated on the plot. Nucleoplasm-enriched (purple), chromatin-enriched (blue), non-enriched (red), and remaining (grey) genes are shown. Right: XY correlation plot of gene expression versus the log2 fold change of “Chromatin RNA-seq versus POINT-seq”. LncRNAs (green) and remaining (grey) genes are shown. **(E)** Correlation matrix heatmap for the mNET-seq showing positive correlation in red and absence of correlation in blue. **(F)** Boxplots, shown as min to max with first quartile, median, and third quartile, of the expression in total pol II mNET-seq of the nucleoplasm-enriched (purple), chromatin-enriched (blue), or non-enriched (red) genes. **(G)** Boxplots, shown as min to max with first quartile, median, and third quartile, of the expression (TPM) in chromatin RNA-seq of the nucleoplasm-enriched (purple), chromatin-enriched (blue), or non-enriched (red) genes. **(H)** XY correlation plot of the log2 fold change of “nucleoplasm RNA-seq versus chromatin RNA-seq” versus the elongation rate from (49)). The Pearson correlation with p-value is indicated on the plot. Nucleoplasm-enriched (purple), chromatin-enriched (blue), non-enriched (red), and remaining (grey) genes are shown.

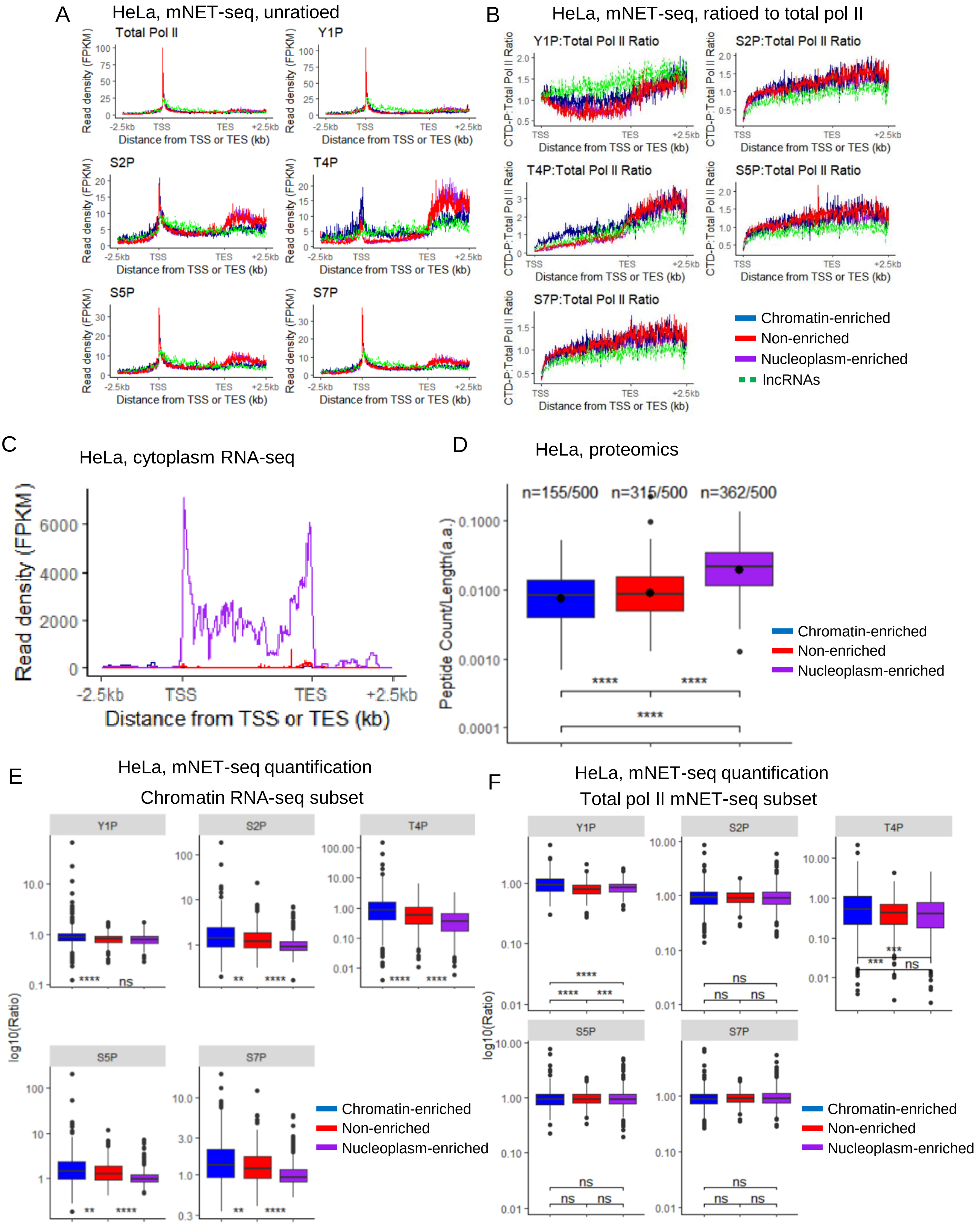

Supplementary Figure 2

Supplementary Figure 2. Pol II CTD phosphorylation levels differ between genes encoding chromatin-enriched or nucleoplasm-enriched transcripts.

(A) Metagene profiles of mNET-seq in HeLa cells of total pol II and the different pol II CTD phosphorylation mark for nucleoplasm-enriched (purple), chromatin-enriched (blue), non-enriched (red) genes, and lncRNAs (green). **(B)** Metagene profiles of mNET-seq in HeLa cells of each pol II CTD phosphorylation mark ratioed to total pol II for nucleoplasm-enriched (purple), chromatin-enriched (blue), non-enriched (red) genes, and lncRNAs (green). **(C)** Metagene profiles in HeLa cells of cytoplasm RNA-seq of the 500 chromatin RNA-seq subsampled nucleoplasm-enriched (purple), chromatin-enriched (blue), or non-enriched (red) genes. **(D)** Boxplots, shown as min to max with first quartile, median, and third quartile, of the number of peptides per protein found for the 500 chromatin RNA-seq subsampled nucleoplasm-enriched (purple), chromatin-enriched (blue), or non-enriched (red) genes. The number of proteins found to have at least one peptide are indicated at the top of each category. Statistical test: Wilcoxon rank sum test. P-value: **** < 0.0001. **(E)** Boxplots, shown as min to max with first quartile, median, and third quartile, of each pol II CTD phosphorylation mark ratioed to total pol II across the gene body of the chromatin RNA-seq selected 500 nucleoplasm-enriched (purple), chromatin-enriched (blue), and non-enriched (red) genes. Statistical test: Wilcoxon rank sum test. P-value: ns: not significant, ** < 0.01, **** < 0.0001. **(F)** Boxplots, shown as min to max with first quartile, median, and third quartile, of each pol II CTD phosphorylation mark ratioed to total pol II across the gene body of the total pol II mNET-seq selected nucleoplasm-enriched (purple), chromatin-enriched (blue), and non-enriched (red) genes. Statistical test: Wilcoxon rank sum test. P-value: ns: not significant, *** < 0.001, **** < 0.0001.

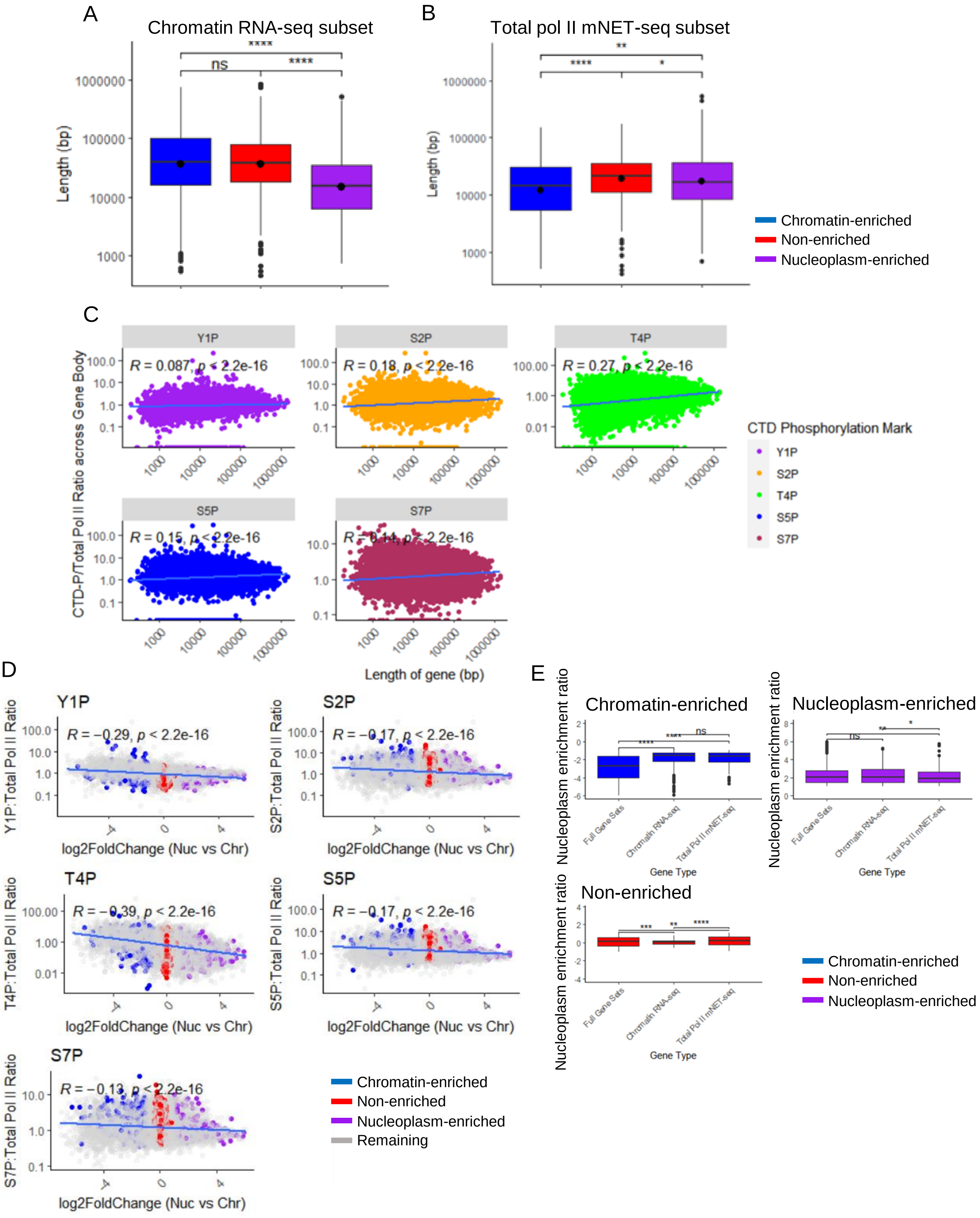

Supplementary Figure 3

Supplementary Figure 3. Gene size does not have any major effect on pol II CTD phosphorylation levels across the gene body.

(A) Boxplots, shown as min to max with first quartile, median, and third quartile, of the gene length of the 500 chromatin RNA-seq selected nucleoplasm-enriched (purple), chromatin-enriched (blue), and non-enriched (red) genes. Statistical test: Wilcoxon rank sum test. P-value: n.s. not significant, **** < 0.0001. **(B)** Boxplots, shown as min to max with first quartile, median, and third quartile, of the gene length of the 10% total pol II mNET-seq selected nucleoplasm-enriched (purple), chromatin-enriched (blue), and non-enriched (red) genes. Statistical test: Wilcoxon rank sum test. P-value: * < 0.05, ** < 0.01, **** < 0.0001. **(C)** XY correlation plots of the gene length (in bp) and each pol II CTD phosphorylation mark ratioed to total pol II. The Pearson correlation with p-value is indicated on each plot. Nucleoplasm-enriched (purple), chromatin-enriched (blue), non-enriched (red), and remaining (grey) genes are shown. **(D)** XY correlation plots of the nucleoplasm fold enrichment, defined as the fold change between nucleoplasm RNA-seq versus chromatin RNA-seq, and each pol II CTD phosphorylation mark ratioed to total pol II. The Pearson correlation with p-value is indicated on each plot. Nucleoplasm-enriched (purple), chromatin-enriched (blue), non-enriched (red), and remaining (grey) genes are shown. **(E)** Boxplots, shown as min to max with first quartile, median, and third quartile, of the nucleoplasm fold enrichment, defined as the fold change between nucleoplasm RNA-seq versus chromatin RNA-seq, for the full set, chromatin RNA-seq subset, and mNET-seq subset of genes for nucleoplasm-enriched (purple), chromatin-enriched (blue), and non-enriched (red) categories.

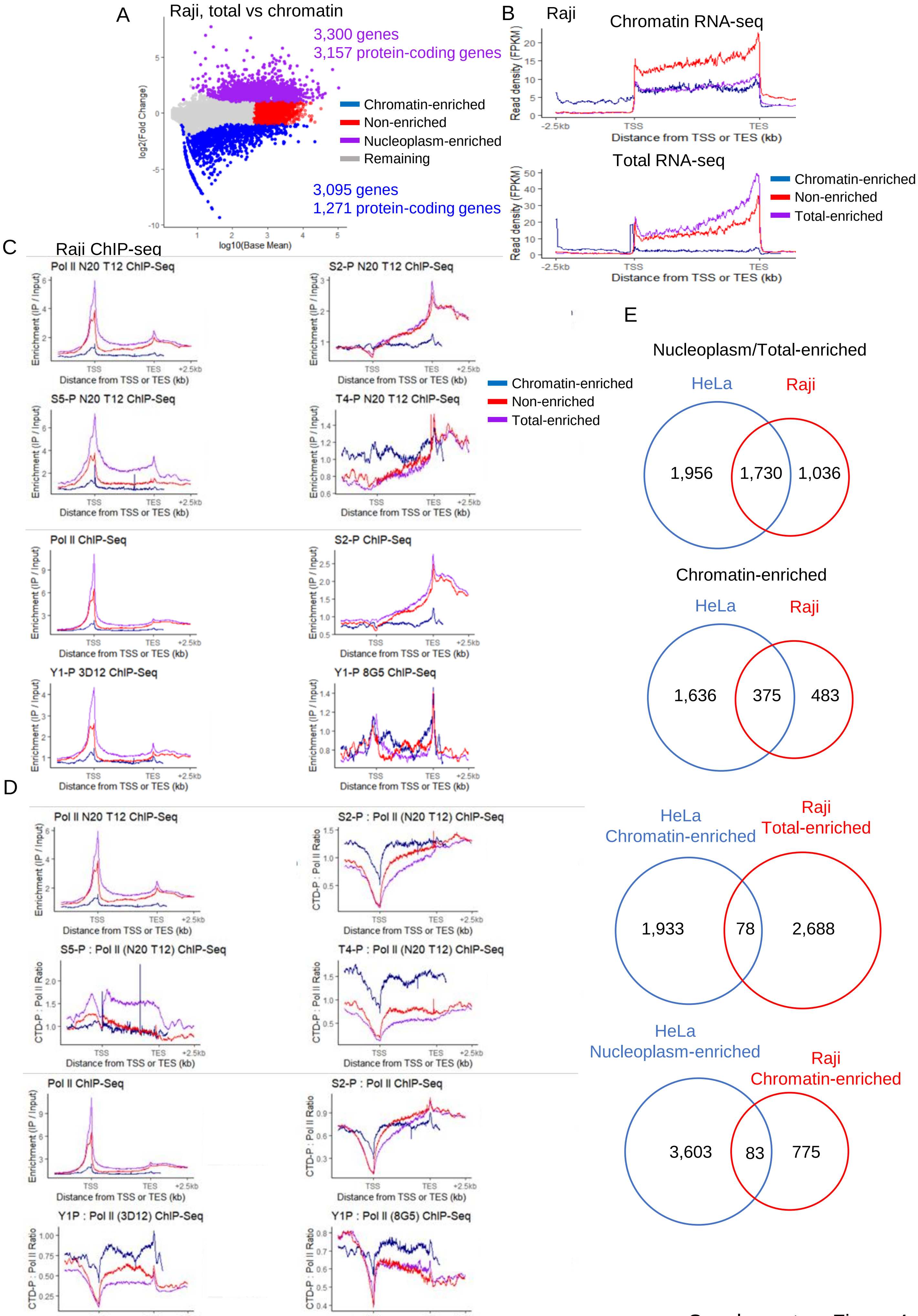

Supplementary Figure 4

Supplementary Figure 4. Raji pol II CTD ChIP-seq show similar patterns to HeLa pol II CTD mNET-seq.

(A) MA plot in Raji cells of the intron-containing protein-coding genes found to be differentially enriched in the total (total-enriched, purple) or in the chromatin (chromatin-enriched, blue) fraction. A set of non-enriched genes (red) and the remaining genes (grey) are also indicated. **(B)** Metagene profiles in Raji cells of chromatin RNA-seq and total RNA-seq of total-enriched (purple), chromatin-enriched (blue), or non-enriched (red) genes. **(C)** Metagene profiles of total pol II, Tyr1P, Ser2P, Thr4P, and Ser5P in Raji cells across the Raji-defined total-enriched (purple), chromatin-enriched (blue), or non-enriched (red) genes. **(D)** Metagene profiles of each Tyr1P, Ser2P, Thr4P, or Ser5P signal ratioed to total pol II signal in Raji cells across the Raji-defined total-enriched (purple), chromatin-enriched (blue), or non-enriched (red) genes. **(E)** Overlap between nucleoplasm/total-enriched genes or between chromatin-enriched genes found to be expressed in both HeLa and Raji cells.

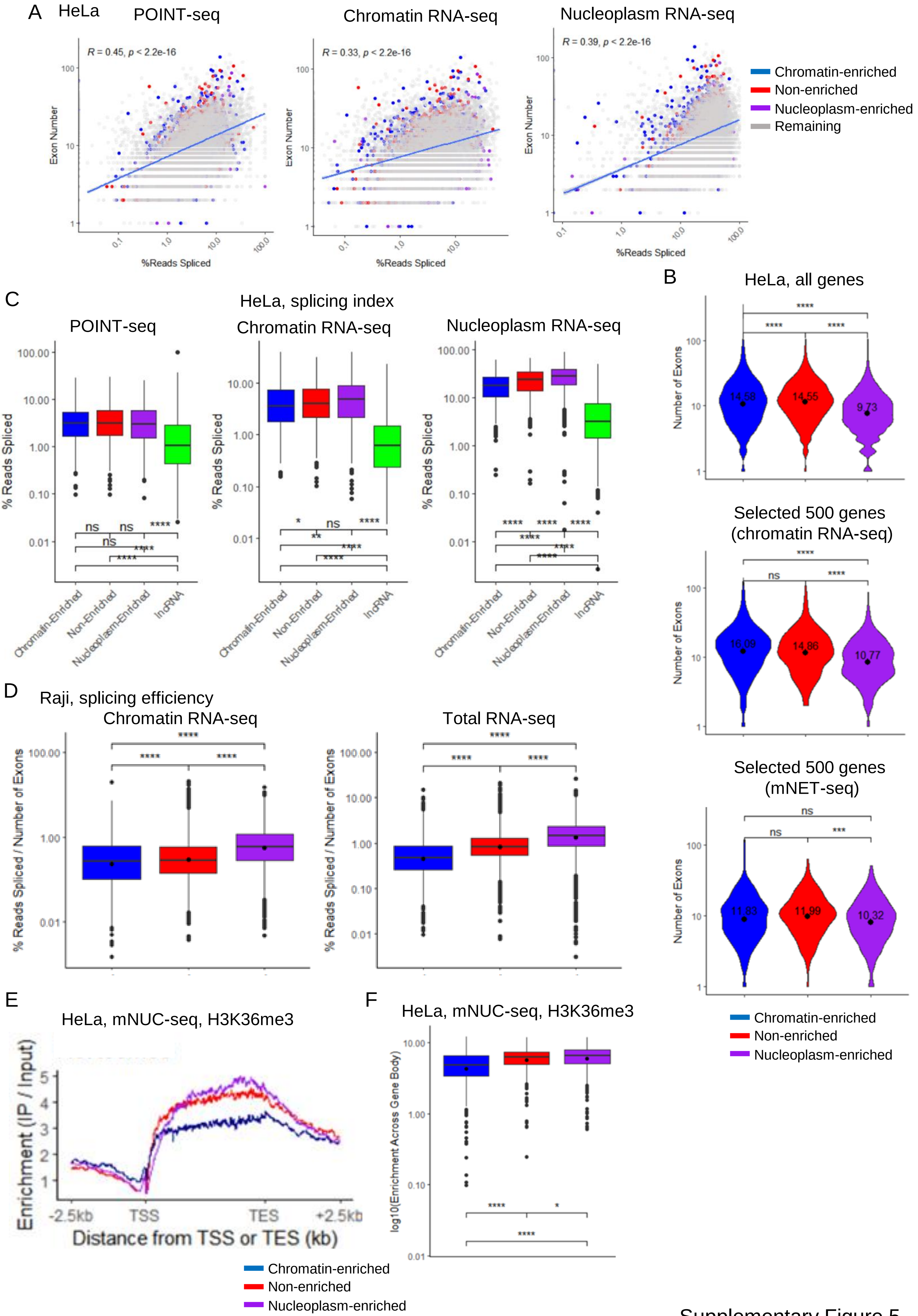

Supplementary Figure 5

Supplementary Figure 5. Transcripts from chromatin-enriched genes are less co-transcriptionally spliced.

(A) XY correlation plots of the splicing efficiency versus the number of exons of each transcript in the POINT-seq, chromatin RNA-seq, and nucleoplasm RNA-seq. The Pearson correlation with p-value is indicated on each plot. Nucleoplasm-enriched (purple), chromatin-enriched (blue), non-enriched (red), and remaining (grey) genes are shown. **(B)** Violin plots, shown as min to max with first quartile, median, and third quartile, of the number of exons in all, or in the chromatin RNA-seq selected 500, nucleoplasm-enriched (purple), chromatin-enriched (blue), non-enriched (red) genes. Statistical test: Wilcoxon rank sum test. P-value: ns: not significant, ** < 0.01, **** < 0.0001. **(C)** Boxplots, shown as min to max with first quartile, median, and third quartile, of the splicing index of each transcript normalized to the number of exons from the POINT-seq, chromatin RNA-seq, and nucleoplasm RNA-seq data in HeLa of the nucleoplasm-enriched (purple), chromatin-enriched (blue), non-enriched (red) genes, and lncRNAs (green). Statistical test: Wilcoxon rank sum test. P-value: ns: not significant, * < 0.05, ** < 0.01, **** < 0.0001. **(D)** Boxplots, shown as min to max with first quartile, median, and third quartile, of the splicing index of each transcript normalized to the number of exons from the chromatin RNA-seq and total RNA-seq data in Raji of the total-enriched (purple), chromatin-enriched (blue), and non-enriched (red) genes. Statistical test: Wilcoxon rank sum test. P-value: **** < 0.0001. **(E)** Metagene profiles in HeLa cells of H3K36me3 mNuc-seq of the nucleoplasm-enriched (purple), chromatin-enriched (blue), non-enriched (red) genes. H3K36me3 is shown across the whole gene body (TSS – 2.5 kb to TES + 2.5 kb). Metaprofiles are shown as IP / Input. **(F)** Boxplots, shown as min to max with first quartile, median, and third quartile, of H3K36me3 ratioed to Input of the nucleoplasm-enriched (purple), chromatin-enriched (blue), non-enriched (red) genes. Quantification is performed on reads located within TSS to TES. Statistical test: Wilcoxon rank sum test. P-value: ns: not significant, * < 0.05, ** < 0.01, **** < 0.0001.

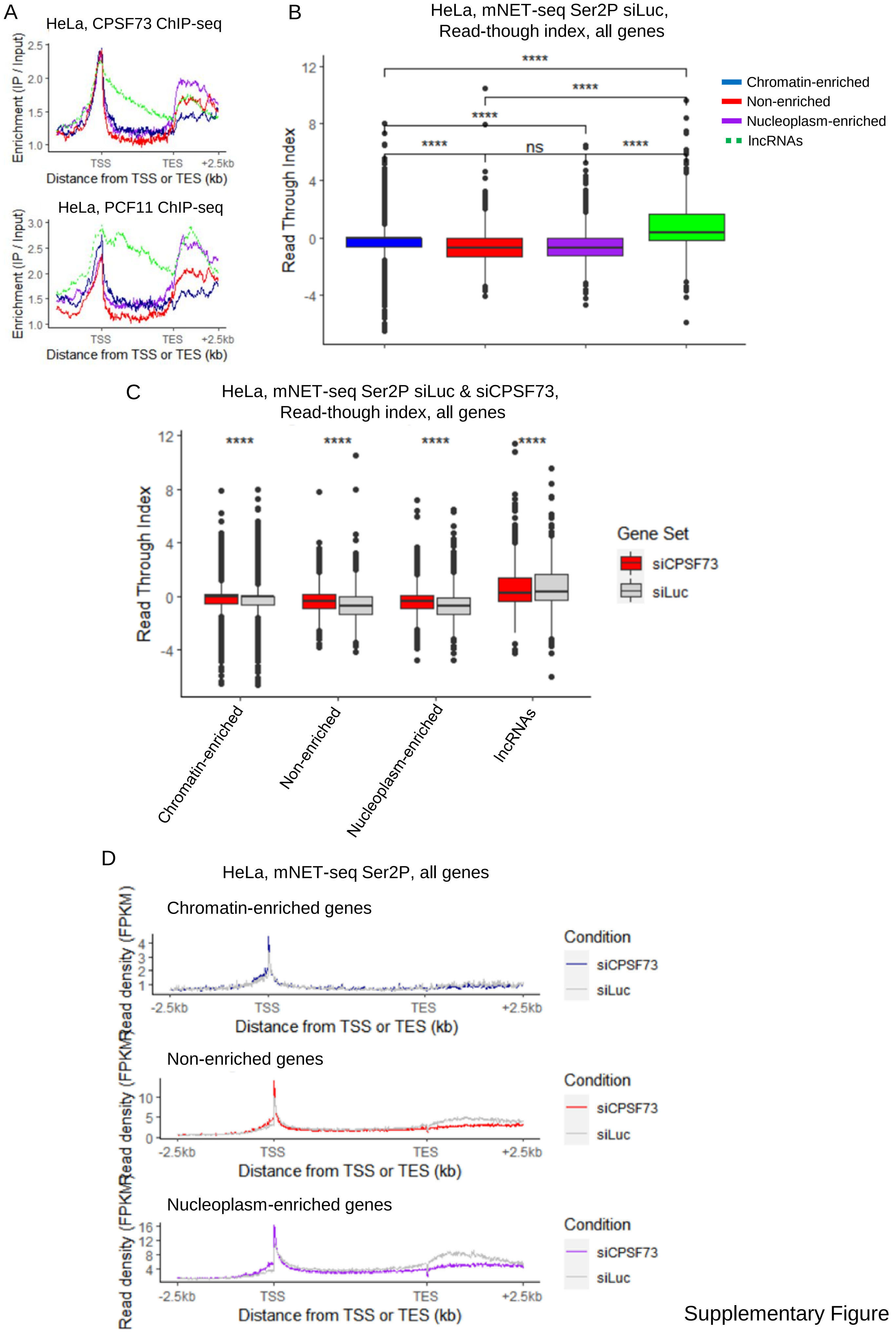

Supplementary Figure 6

Supplementary Figure 6. Transcripts from chromatin-enriched genes have a weak mRNA cleavage and polyadenylation.

(A) Metagene profiles in HeLa cells of CPSF73 and PCF11 ChIP-seq of all nucleoplasm-enriched (purple), chromatin-enriched (blue), non-enriched (red) genes, or lncRNAs (green). **(B)** Boxplots, shown as min to max with first quartile, median, and third quartile, of the read-through index calculated on the Ser2P mNET-seq treated with siLuc of all nucleoplasm-enriched (purple), chromatin-enriched (blue), non-enriched (red) genes, or lncRNAs (green). Statistical test: Wilcoxon rank sum test. P-value: ns: not significant, **** < 0.0001. **(C)** Boxplots, shown as min to max with first quartile, median, and third quartile, of the read-through index calculated on the Ser2P mNET-seq treated with siLuc (grey) or siCPSF73 (red) of all nucleoplasm-enriched, chromatin-enriched, non-enriched genes, or lncRNAs. Statistical test: Wilcoxon rank sum test. P-value: **** < 0.0001. **(D)** Metagene profiles in HeLa cells of chromatin RNA-seq treated with siLuc (black) or siCPSF73 (blue) of all nucleoplasm-enriched, chromatin-enriched, or non-enriched genes.

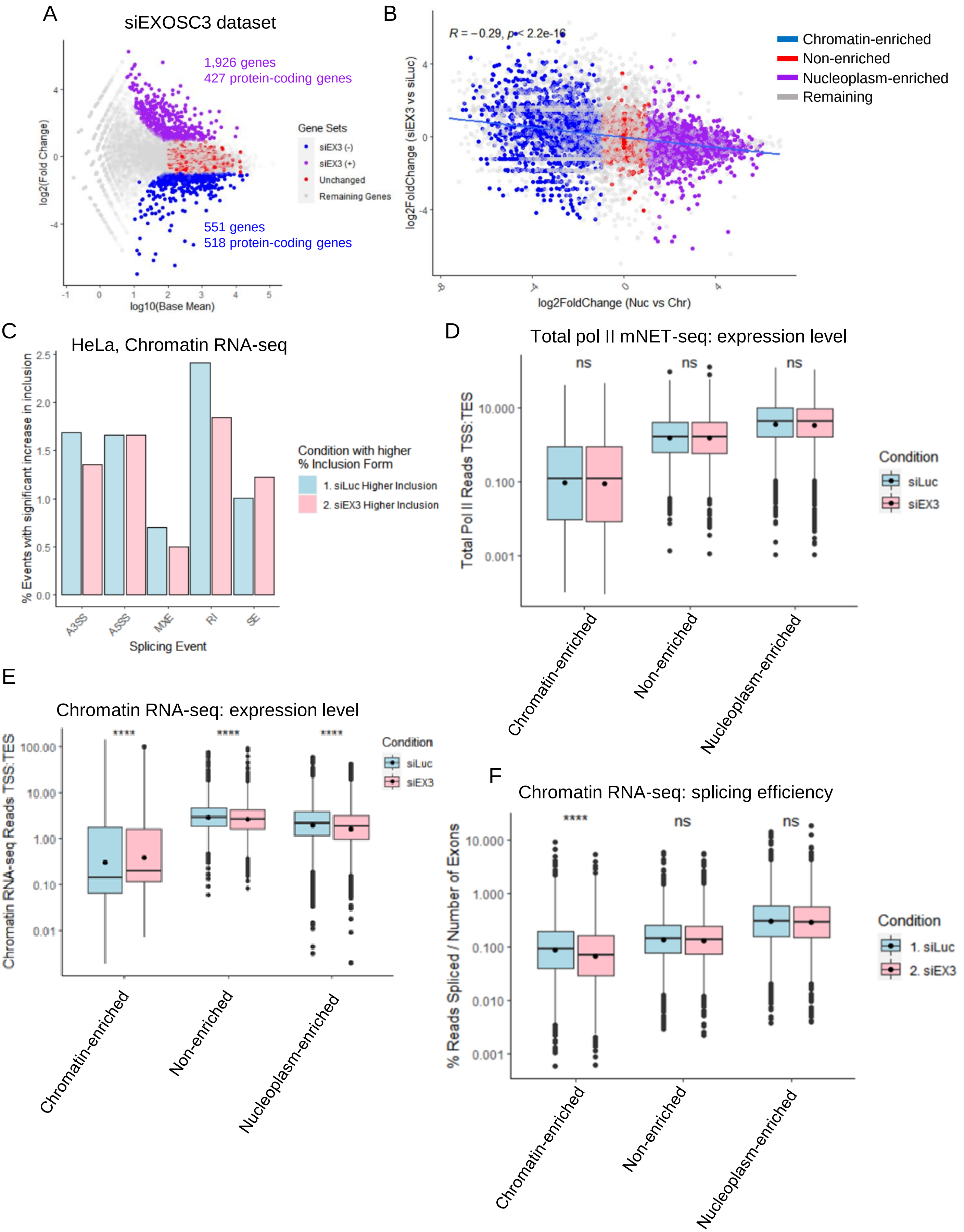

Supplementary Figure 7

Supplementary Figure 7. Transcripts from chromatin-enriched gene are sensitive to the nuclear RNA exosome.

(A) MA plot in HeLa cells of the intron-containing protein-coding genes found to be upregulated (siEX3(+), purple) or downregulated (siEX3(-), blue) after siEXOSC3. A set of non-enriched genes (red) and the remaining genes (grey) are also indicated. **(B)** XY correlation plot of the nucleoplasm fold enrichment, defined as the log2 fold change between nucleoplasm RNA-seq versus chromatin RNA-seq, and the log2 fold change in siEXOSC3 versus siLuc. Transcripts from chromatin-enriched, non-enriched, and nucleoplasm-enriched genes are shown in blue, red, and purple, respectively. The Pearson correlation with p-value is indicated on the plot. **(C)** Bar charts of significant changes in chromatin RNA-seq of splicing events obtained with rMATs in control (siLuc, blue) or following the knockdown of the nuclear RNA exosome (siEX3, pink). A3SS: alternative 3' splice site; A5SS: alternative 5' splice site, MXE: mutually exclusive exons; IR: intron retention; SE: skipped exon. **(D) and (E)** Boxplots, shown as min to max with first quartile, median, and third quartile, of the expression level of full-length transcripts, including exons and introns, from the total pol II mNET-seq **(D)** and chromatin RNA-seq **(E)** data in HeLa cells in control (siLuc, blue) or after siEXOSC3 knockdown (siEX3, pink) of all nucleoplasm-enriched, chromatin-enriched, or non-enriched genes. Statistical test: Wilcoxon rank sum test. P-value: n.s. not significant, **** < 0.0001. **(F)** Boxplots, shown as min to max with first quartile, median, and third quartile, of the splicing index of each transcript normalized to the number of exons from the chromatin RNA-seq data in HeLa cells in control (siLuc, blue) or after siEXOSC3 knockdown (siEX3, pink) of all nucleoplasm-enriched, chromatin-enriched, or non-enriched genes. Statistical test: Wilcoxon rank sum test. P-value: n.s. not significant, **** < 0.0001.
